# The effect of training methodology on knowledge representation in categorization

**DOI:** 10.1371/journal.pone.0183904

**Published:** 2017-08-28

**Authors:** Sébastien Hélie, Farzin Shamloo, Shawn W. Ell

**Affiliations:** 1 Department of Psychological Sciences, Purdue University, West Lafayette, Indiana, United States of America; 2 Department of Psychology, University of Maine, Orono, Maine, United States of America; Waseda University, JAPAN

## Abstract

Category representations can be broadly classified as containing within–category information or between–category information. Although such representational differences can have a profound impact on decision–making, relatively little is known about the factors contributing to the development and generalizability of different types of category representations. These issues are addressed by investigating the impact of training methodology and category structures using a traditional empirical approach as well as the novel adaptation of computational modeling techniques from the machine learning literature. Experiment 1 focused on rule–based (RB) category structures thought to promote between–category representations. Participants learned two sets of two categories during training and were subsequently tested on a novel categorization problem using the training categories. Classification training resulted in a bias toward between–category representations whereas concept training resulted in a bias toward within–category representations. Experiment 2 focused on information-integration (II) category structures thought to promote within–category representations. With II structures, there was a bias toward within–category representations regardless of training methodology. Furthermore, in both experiments, computational modeling suggests that only within–category representations could support generalization during the test phase. These data suggest that within–category representations may be dominant and more robust for supporting the reconfiguration of current knowledge to support generalization.

## Introduction

Categorical representations (i.e., the way in which information is stored and used [1]) are the building blocks of decision–making from the most routine to the most novel contexts [2]. Not surprisingly, the study of the processes underlying the development of the representations necessary for such decision making has been the focus of much research [3–6]. Researchers advocating for one theory or another have tended to focus on a single paradigm, suggesting that some theoretical disagreements may be driven by methodological differences.

However, generally speaking, category representations can be broadly classified as within–category representations or between–category representations [5, 7]. Specifically, within–category representations contain information about the categories themselves. For example, a within–category representation of *humans* could contain information about what is common among category members (e.g., one head), the correlation between the features (e.g., as height increases, so does arm length), or the range of feature values (e.g., adult height typically varies between 5–6.5 feet). In contrast, between–category representations would contain information about the distinguishing features between two categories. For example, a between–category representation contrasting *humans* and *dogs* might contain information about the relevant features for separating humans and dogs (e.g., number of legs) and criteria on these feature–values (e.g., less than three legs is generally a human; more than three legs is generally a dog). Generally, within–category representations may be more useful when inferring missing attributes (e.g., one may infer that my spouse has two legs without being told) whereas between–category representations may be useful for extrapolation outside of the learning space (e.g., an animal with one leg is even less likely to be a dog than a human). In the category learning literature, prototype models [6, 8] and exemplar models [9] assume that a within–category representation is the basis for response selection, whereas criterion–setting models [10, 11] assume a between-category representation.

At this point, however, there is only limited understanding of the factors influencing the development of various category representations [12]. Furthermore, it is even less clear if different types of category representations vary in their generalizability. The goal of this article is to explore the factors contributing to the development and generalizability of different types of category representations. In particular, this article focuses on the impact of training methodology and category structure on category representation. In addition, we employ an interdisciplinary approach by utilizing computational techniques from the machine learning literature to analyze individual differences in category representation.

### Knowledge representation in category learning

Task goal (i.e., the task procedures necessary for successful performance) has been identified as a factor that can influence the type of category representations [5]. For instance, in a typical category learning experiment, participants are presented with a number of stimuli and instructed to make a decision about category membership for each one (e.g., classifying a bug–like stimulus with red markings, a round head, and long legs as a member of category “A” or “B”). It has been argued that such classification instructions lead to the development of between–category representations (e.g., learn what dimensions are relevant for classification, along with decision criteria or category boundaries, e.g., [3, 4, 6, 13]). In contrast, inference tasks, in which participants are presented with an incomplete stimulus along with a category label and asked to fill–in the missing stimulus feature, have been shown to lead to the development of within–category representations (e.g., the correlation between the stimulus–features).

Although the task goal of classification may be a necessary condition for the development of between–category representations, it does not appear to be sufficient. For instance, a variation on the typical classification instructions emphasizes concept learning (i.e., participants learn categories by classifying stimuli as a member or non–member of a target category, e.g., [6, 14–17]). Recent work suggests that this may not be a trivial distinction because concept and classification training may lead to different psychological representations [18].

In addition, Carvalho and Goldstone have shown that stimulus ordering is also relevant [19]. Specifically, increasing the probability that the stimulus presented in trial *t* + 1 is from the same category as the stimulus presented in trial *t* (a condition called *blocked*) results in participants learning within–category representations whereas alternating between different categories from trial to trial (a condition called *interleaved*) results in participants learning between–category representations. Carvalho and Goldstone argued that learning is driven by an attentional mechanism comparing the current stimulus with the previous stimulus [19]. If the two stimuli are from the same category, then the participant learns the commonalities between these stimuli (i.e., within–category representation). If the stimuli are from different categories, then the participant learns what distinguishes the stimuli (i.e., between–category representation).

Similarly, in the machine learning literature, different computational techniques have been designed for classification versus concept training. Interestingly, algorithms used to perform optimally in classification tasks (e.g., pattern classifiers [20]) generally learn between–category representations (e.g., separation planes). In contrast, algorithms used to perform optimally in concept learning tasks (e.g., inductive logic programming [21]) generally learn within–category representations (e.g., generative models). Thus, both machine learning and cognitive psychology converge on the prediction that classification and concept training should fundamentally alter the way in which categorical information is stored and used.

The structure of the categories themselves can also influence the category representation, e.g., rule–based (RB) versus information–integration (II) [22]. RB structures are those in which the categories can be learned using logical rules. Although logical rules can be based on either within– or between–category representations (e.g., large vs. larger than), the subset of logical rules learned in RB tasks tends to depend upon between–category representations [23–25]. For example, the hypothesis–testing system in COVIS [3], used to learn RB structures, is a criterion setting model that learns a between-category representation. In contrast, II category structures are those in which information from multiple dimensions needs to be integrated prior to making a categorization response. Unlike RB structures, II structures may promote within–category representations [23, 26, 27]. For example, the procedural learning mechanism in COVIS (used to learn II structures) is a radial–basis function connectionist network (computationally equivalent to a Gaussian mixture model, which is a generative model) that learns a within-category representation. Importantly, despite the task goal being identical (e.g., classification), neurocomputational models that have been applied to RB and II structures implicitly echo the hypothesis that RB structures promote between–category representations whereas II structures promote within–category representations [3, 28, 29].

### The current experiments

Taken together, the aforementioned evidence from cognitive psychology and machine learning research lead to the hypothesis that both task goal and category structure may be important factors in determining whether individuals learn between– or within–category representations. Both the task goal of classification and the learning of RB structures would generally be expected to lead to a between–category representation. However, we propose that this representational bias can be overcome in two important ways. First, concept training can overcome the apparent inevitability of between–category representations in RB structures. Optimal models of concept learning depend upon within–category representations [21]. Thus, to the extent that performance is rational [30], concept training would be predicted to shift the representational bias toward within–category representations. Second, II structures promote within–category representations (e.g., [26]) and, therefore, will overcome the apparent inevitability of between-category representations with classification training.

It is also important to note that classification is but one of the many uses of category representations [2, 5, 31]. For example, an animal does not classify a plant as edible because it enjoys the intellectual challenge; it makes the categorization because it needs to eat. In this example, categorization serves as an intermediate step to facilitate the interaction of the animal with the object. Importantly, we argue that the generalizability of category representations depends upon the nature of the representation itself. For instance, between–category representations may be better suited to generalize to novel stimuli that are outside of the previously encountered perceptual space (e.g., because the representation is not tied to the stimuli themselves, but rather between–category differences [32]). Similarly, within–category representations may be better suited to generalization that would benefit from knowledge of within–category regularities, such as prototypicality or the covariation of stimulus dimensions [33].

To test these hypotheses, we contrast two classification and concept training paradigms. For classification training, participants were instructed to distinguish between members of contrasting categories (e.g., Is the stimulus a member of category A or category B?—hereafter referred to as A/B training). For concept training, participants were instructed to distinguish between category members and non–members (e.g., Is the stimulus a member of category A?—hereafter referred to as Yes/No training) [14]. Participants were trained separately on two sets of two categories (categories A and B; categories C and D—for a total of four categories) and subsequently completed a test phase using a novel combination of the training categories (i.e., categories B and C). Learning within–category representations should allow for successful generalization during the test phase because participants would have knowledge of within-category similarities for all four categories. In contrast, learning between–category representations should limit generalization during the test phase because participants’ knowledge would be limited to the specific between–category differences emphasized during training. Thus, for RB structures (Experiment 1), we hypothesize that A/B training would result in between–category representations, but Yes/No training should shift the bias towards within–category representations. In contrast, for II structures (Experiment 2), we hypothesize a bias towards within–category representations regardless of training methodology.

An additional contribution of the current experiments is the development of a computational method that can identify the type of representations learned by individual participants (i.e., within– vs. between–category). We adapt a technique from the machine learning literature in which models are fit to the training data, but evaluated on the test data (i.e., cross–validation [34, 35], see also [36]). Rather than focus on some subset of the numerous published categorization models, we take the approach of using more general computational models as a tool to test classes of statistical models [20]. Specifically, a linear classifier is used to represent the class of models assuming a between–category representation (e.g., criterion-setting models [10]) and a Gaussian density model is used to represent the class of models assuming a within–category representation (e.g., prototype models [8]). Importantly, the computational model in this article is not meant to be a new contender in the world of categorization models, but instead a framework for testing classes of models with common underlying assumptions about the nature of category representation (namely within– or between–category representation).

To anticipate, the results show that with RB structures (Experiment 1), participants were more likely to learn within–category representations with Yes/No training than with A/B training. In contrast, training methodology had no effect on representations when learning II structures (Experiment 2), and most participants learned within–category representations. As a result, participants in A/B training of Experiment 1 were not able to generalize their knowledge in the test phase (because within–category representations are necessary at the test phase). In addition, the type of category representations identified using the new computational method correctly predicts the presence or absence of transfer for each participant in each condition in both experiments.

## Experiment 1

The goal of Experiment 1 was to test the hypothesis that training methodology affects the nature of, and the ability to generalize, category representations learned with RB structures. Specifically, participants were trained on four categories using either concept training instructions (Yes/No) or classification training (A/B) instructions. Importantly, the stimuli and categories were the same in both training conditions. In both cases, the training phase asked participants to differentiate between two sets of two categories (i.e., A vs. B and C vs. D), and the test phase asked participants to make a novel categorization decision using two of the categories learned during the training phase (i.e., B vs. C). If the participants learned within–category representations of categories B and C during training, generalizing this knowledge should make them well–equipped to distinguish between categories B and C during the test phase. However, if the participants learned between–category representations, generalizing this knowledge should make them poorly equipped to distinguish between categories B and C during the test phase because the training phase did not require participants to learn what makes category B different from category C.

### Method

#### Participants

Sixty-one participants were recruited from large state universities to participate in this experiment. Participants were randomly assigned to one of two training conditions: Yes/No (*n* = 31) and A/B (*n* = 30). Each participant gave informed written consent and received credit for participation as partial fulfillment of a course requirement. All procedures were approved by the Purdue University Social Science Institutional Review Board and the University of California Santa Barbara Human Subjects Committee.

#### Material

The stimuli were circular sine–wave gratings of constant contrast and size presented on a 21–inch monitor (1,280 × 1,024 resolution). Each stimulus was defined in a 2D space by a set of points (*frequency*, *orientation*) where *frequency* (bar width) was calculated in cycles per degree (cpd), and *orientation* (counterclockwise rotation from horizontal) was calculated in radians. The stimuli were generated with Matlab using the Psychophysics toolbox [37] and occupied an approximate visual angle of 5°. In each trial, a single stimulus was presented in the center of the screen.

**Fig 1:**
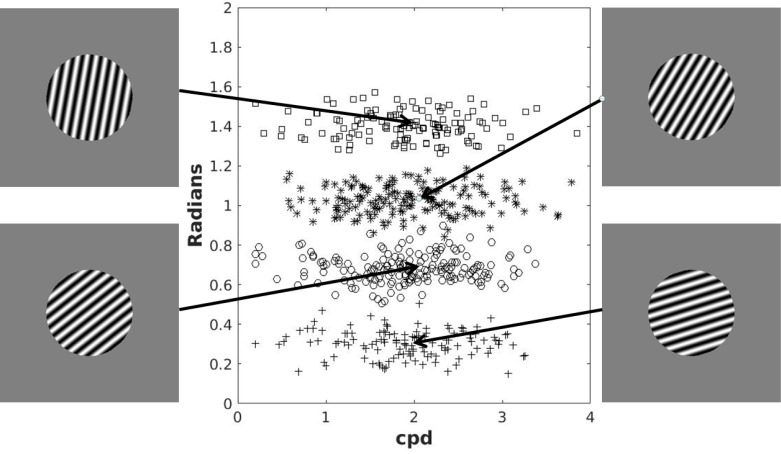
Stimuli used in Experiment 1. The *x*-axis corresponds to the width of the bars and the *y*-axis corresponds to the rotation angle of the bars. Symbols denote different categories. The mean stimulus of each category is shown as an example.

The categories used for both instruction conditions are shown in Fig 1. There were four separate categories generated using the randomization technique [38]. The categories were arbitrarily labeled with letters A-D from bottom to top. Category A stimuli were generated using a bivariate normal distribution with mean *μ*_*A*_ = (1.9, 0.30) and covariance $\Sigma_{A}=(\begin{array}{cc} 0.44 & 0\\ 0 & 0.01 \end{array})$. Category B stimuli were generated using a bivariate normal distribution with mean *μ*_*B*_ = (1.9, 0.67) and covariance Σ_*B*_ = Σ_*A*_. Category C stimuli were generated using a bivariate normal distribution with mean *μ*_*C*_ = (1.9, 1.03) and covariance Σ_*C*_ = Σ_*A*_. Category D stimuli were generated using a bivariate normal distribution with mean *μ*_*D*_ = (1.9, 1.40) and covariance Σ_*D*_ = Σ_*A*_. This yielded stimuli that varied in orientation from 10° to 90° (counterclockwise from horizontal) and in bar width (frequency) between 0.2 and 3.85 cpd. These categories can be near–perfectly separated by three linear boundaries corresponding to the following verbal rule: near–horizontal stimuli are “A”, slightly steeper stimuli are “B”, much steeper stimuli are “C”, and near vertical stimuli are “D”. A single set of 600 stimuli was generated from these distributions (shown in Fig 1), and the stimulus set was linearly transformed so that the sample mean and covariance of each category matched the generative distributions. The stimulus set was independently shuffled for each participant.

Stimulus presentation, feedback, and response recording were controlled and acquired using Matlab. Responses were given on a standard keyboard. In the A/B condition, the following question was displayed in the top–middle of the screen in black font “X or Y?” where X and Y were replaced by category labels informing the participants that they should respond using one of these two categories in this trial. The participants responded “A” using the “d” key, “B” using the “k” key, “C” using the “x” key, and “D” using the “m” key (sticker labeled). In the Yes/No condition, the following question was displayed in the top–middle of the screen in black font “Is this a ‘X’?” where X was replaced by a category label informing the participants of the target category for this trial. The participants responded “yes” using the “d” key and “no” using the “k” key (sticker labeled). In both conditions, visual feedback was given for a correct (the word “Correct” in green font) or incorrect (the word “Incorrect” in red font) response. If a response was too late (more than 5 seconds), participants saw the words “Too Slow” in black font. If a participant hit a wrong key, the words “Wrong Key” were displayed in black font. During the whole experiment, the screen background was gray.

#### Procedure

The experiment was composed of 6 blocks of 100 trials (for a total of 600 trials), and each stimulus was seen only once. Participants were told they were taking part in a categorization experiment and that they had to assign each stimulus into either an “A”, “B”, “C”, or “D” category. The participants were told that there would be a test phase at the end of the experiment, but they were not told what the test was until they finished the training phase.

The first 5 blocks were training blocks and the participants were trained to separate “A” stimuli from “B” stimuli and “C” stimuli from “D” stimuli. Specifically, only the questions “A or B?” and “C or D?” were used in the A/B condition. Likewise, if the question was “Is this an ‘A’?” in the Yes/No condition, “no” responses were from the “B” category, and if the question was “Is this a ‘B’?”, “no” responses were from the “A” category (and the same logic applies to the “C” and “D” categories). All categories and questions were equally likely. After the training phase, the participant were told that they were now beginning the test phase, and that they should use the categories learned during the training phase to respond in the test phase. They were also told that they would no longer be receiving feedback. Note that the training and test stimulus sets were non–overlapping and randomly selected for each participant.

**Fig 2:**
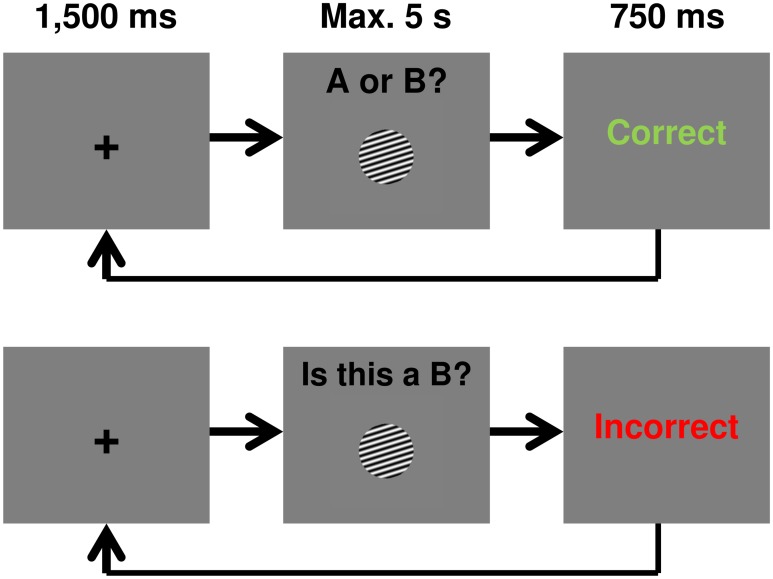
Experimental procedures used in both experiments. The top line shows an example A/B training trial while the bottom line shows an example Yes/No training trial. Test trials were identical except that feedback was omitted.

A training trial went as follows: a fixation point (crosshair) appeared on the screen for 1,500 ms and was followed by the stimulus and the question. The question and stimulus remained on the screen until the participant made a response. When the participants made a response or after 5 seconds has elapsed, the stimulus and question disappeared and feedback was presented for 750 ms. A schematic showing a training trial in each condition is shown in Fig 2. The participants were allowed to take a break between blocks if they wished.

The test phase (block 6) was identical to the training blocks except that participants were now asked to separate “B” stimuli from “C” stimuli. Specifically, the question “B or C?” was used in every test trial of the A/B condition. In the Yes/No condition, test trials used the questions “Is this a ‘B’?” or “Is this a ‘C’?”, and “no” responses were always from the “C” or “B” categories (respectively). Categories and questions were equally likely. No feedback was provided during the test phase.

### Results

**Fig 3:**
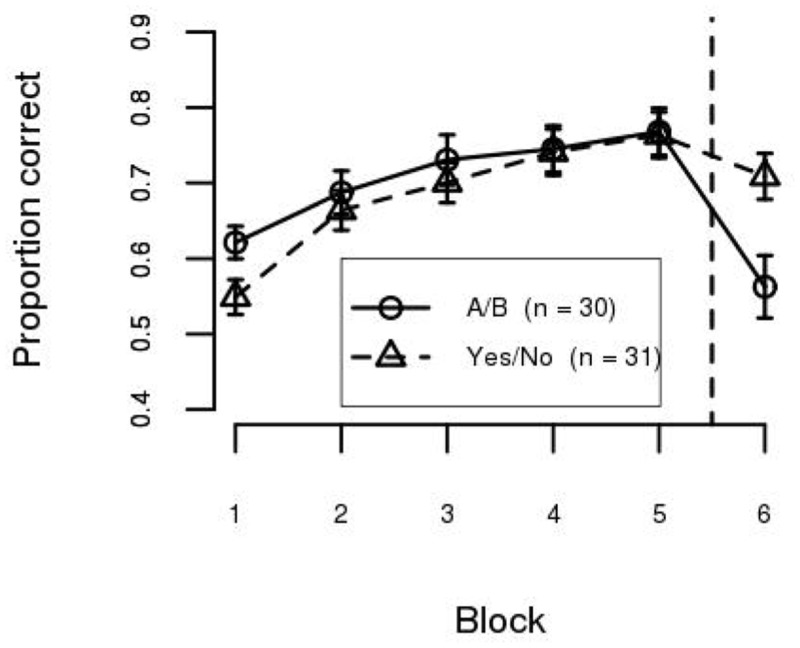
Mean accuracy per block in Experiment 1. Blocks 1-5 are the training phase and Block 6 is the test phase. Error bars are between–subject standard error of the mean.

The mean accuracy for each block in each condition is shown in Fig 3. As can be seen, training accuracy was similar for the A/B and Yes/No training conditions. This was confirmed by a Condition (A/B, Yes/No) × Training Block (1…5) mixed ANOVA. The effect of Block was statistically significant (*F*(4, 236) = 41.50, *p* < .001, *η*^2^ = 0.59) but the effect of Condition (*F*(1, 59) = 0.59, *n*.*s*., *η*^2^ = 0.01) and the interaction between the factors (*F*(4, 236) = 1.60, *n*.*s*., *η*^2^ = 0.08) were not. The mean accuracy on Block 1 was 58.5%, which improved to 76.6% in Block 5, showing that participants in both instruction conditions learned the task at the same rate.

Test phase accuracy, however, differed between the two training conditions. Categorical knowledge was better transferred in the Yes/No condition than in the A/B condition. This was confirmed by a Condition (A/B, Yes/No) × Block (Last training block, Test block) mixed ANOVA. The effect of Block reached statistical significance (*F*(1, 59) = 31.42, *p* < .001, *η*^2^ = 0.35) while the effect of Condition did not (*F*(1, 59) = 2.91, *n*.*s*., *η*^2^ = 0.05). However, these effects need to be interpreted in the context of a statistically significant interaction (*F*(1, 59) = 10.51, *p* < .01, *η*^2^ = 0.15). We decomposed the effect of Block within each level of Condition, and the results show a statistically significant cost in performance at test for both the A/B (*t*(29) = 4.97, *p* < 1 × 10^−4^, *η*^2^ = 0.46) and Yes/No (*t*(30) = 2.48, *p* < .05, *η*^2^ = 0.17) conditions. Mean accuracy in the test phase was 56.3% in the A/B condition (a decrease of 20.5% in accuracy) and 70.9% in the Yes/No condition (a decrease of 5.5% in accuracy). Test accuracy was significantly higher in the Yes/No condition than in the A/B condition (*t*(54) = 2.84, *p* < .01, *η*^2^ = 0.13), indicating that generalization was superior with concept training.

#### Model–based analyses

One useful way of identifying the kind of representation learned by participants is to fit models to individual participants’ performances [39]. The manipulation in Experiment 1 was designed to influence the participants to learn either a within–category representation (e.g., what is common to “A” category members) or a between–category representation (e.g., what is different between “A” and “B” category members). We propose fitting Gaussian density models (e.g., [20]) to capture within–category representations and linear boundary–based models (e.g., [4]) to capture between–category representations. These models have been selected because the optimal bounds separating the categories used in the experiments are linear, and the optimal bound separating two Gaussian distributions is also linear—so both models are optimal for the categories used.

The first step is to draw the participant’s decision space. This means assigning a participant response to each coordinate point in the stimulus space (so that each participant has their own decision space). The result is similar to Fig 1 except that the participant’s responses are used as symbols instead of the desired category labels. For the density–based model, a different 2–dimensional Gaussian distribution is fit to each possible response category (i.e., A–D). For Yes/No training, only trials in which the participant responded “yes” were included, because there is no way to know what category the participant had in mind when responding “no”. For example, the “A” density is estimated by including only trials in which the question was “Is this an ‘A’?” and the participant responded “yes”. The same procedure was used to estimate the densities representing categories B, C, and D. For A/B training, all trials in which the participant pressed the “A” response button were used to estimate the “A” density (or “B”, or “C”, or “D” to estimate the densities corresponding to categories B–D). In all cases, the maximum likelihood estimators were used (i.e., the sample mean and variance). Note that this model has 8 free parameters, i.e., the mean and variance of the radians for each category.

With the density model, the probability of identifying a stimulus as being located in the perceptual region associated with *C*_*X*_ is:

$$\begin{array}{c} p\left(C_{X}|d_{i}\right)=\frac{f_{X}\left(d_{i}\right)}{f_{X}\left(d_{i}\right)+f_{Y}\left(d_{i}\right)} \end{array}$$ (1)

where *p*(*C*_*X*_|*d*_*i*_) is the probability of locating stimulus *d*_*i*_ in the perceptual region associated with category *X* (denoted *C*_*X*_), *f*_*X*_(*d*_*i*_) is the probability of *d*_*i*_ according to the density estimated for *C*_*X*_, and *f*_*Y*_(*d*_*i*_) is the probability of *d*_*i*_ according to the density estimated for *C*_*Y*_, where *Y* is the contrasting category. With the density–based model, Eq 1 is used both at training and at test.

For the boundary–based models, the procedure is similar to that of density–based models except that boundaries are estimated *between* the categories (instead of estimating densities *within* the categories). The procedure is exactly the same as described in [4]. Each bound is represented by a 1–dimensional Gaussian distribution where the mean is the location of the bound and the variance corresponds to perceptual noise. The same participant’s space as for the density–based model is drawn. Because the training phase only contrasted category “A” with “B” and category “C” with “D”, only these two bounds were estimated. Estimating the AB boundary used all the trials in which the question referred to these categories for the Yes/No condition, and all the trials in which response buttons “A” or “B” were pressed for the A/B condition. The same procedure was used to estimate the CD boundary. All the parameters were estimated using maximum likelihood [4]. Note that this model has 4 free parameters, corresponding to the location and noise of each bound.

With the boundary–based model, the probability of identifying a stimulus as being located on the *A* side of the AB bound during training is:

$$\begin{array}{c} p\left(C_{A}|d_{i}\right)=1-F_{AB}\left(d_{i}\right) \end{array}$$ (2)

where *p*(*C*_*A*_|*d*_*i*_) is the probability of locating stimulus *d*_*i*_ on the *A* side of the AB bound (denoted *C*_*A*_), and *F*_*AB*_(*d*_*i*_) is the probability of *d*_*i*_ according to the cumulative density function estimated for the AB bound. The probability of identifying a stimulus as being located on the *B* side of the AB bound is simply *p*(*C*_*B*_|*d*_*i*_) = 1 − *p*(*C*_*A*_|*d*_*i*_) = *F*_*AB*_(*d*_*i*_). The same equation applies at training for the CD bound (but substitute *C*_*C*_ → *C*_*A*_ and *C*_*D*_ → *C*_*B*_).

A different equation needs to be used at test with the boundary–based model because there is no BC bound, and both the AB and the CD bounds need to be considered in identifying a stimulus as being located in the *C*_*B*_ or *C*_*C*_ region. The boundary–based model at test is:

$$\begin{array}{c} p\left(C_{B}|d_{i}\right)=\frac{F_{AB}\left(d_{i}\right)}{\left(1-F_{CD}\left(d_{i}\right)\right)+F_{AB}\left(d_{i}\right)} \end{array}$$ (3)

where *p*(*C*_*B*_|*d*_*i*_) is the probability of locating stimulus *d*_*i*_ in the *C*_*B*_ region at test, *F*_*AB*_(*d*_*i*_) is the probability of *d*_*i*_ according to the cumulative density function estimated at training for the AB bound, and *F*_*CD*_(*d*_*i*_) is the probability of *d*_*i*_ according to the cumulative density function estimated at training for the CD bound. Intuitively, the numerator corresponds to probability of responding B according to the AB bound, and the denominator normalizes according to the probability of responding C according to the CD bound. This extra step is necessary because the AB bound does not allow for calculating the probability of responding C, and the CD bound does not allow for calculating the probability of responding B. The probability of identifying a stimulus as being located in the *C*_*C*_ region at test is simply *p*(*C*_*C*_|*d*_*i*_) = 1 − *p*(*C*_*B*_|*d*_*i*_).

Both the density and boundary models use a common decision function that is described by:

$$\begin{array}{c} p\left(R_{X}|d_{i}\right)=\frac{e^{\alpha p(C_{X}|d_{i})}}{e^{\alpha p(C_{X}|d_{i})}+e^{\alpha p(C_{Y}|d_{i})}} \end{array}$$ (4)

where *p*(*R*_*X*_|*d*_*i*_) is the probability of responding category *X* (denoted *R*_*X*_) when stimulus *d*_*i*_ is present, *p*(*C*_*X*_|*d*_*i*_) is the probability of locating stimulus *d*_*i*_ in the *C*_*X*_ region (as calculated by Eqs 1, 2 or 3), and *α* is a noise parameter estimated by minimizing the sum of square errors (SSE). The probability of responding category *R*_*Y*_ is simply given by *p*(*R*_*Y*_|*d*_*i*_) = 1 − *p*(*R*_*X*_|*d*_*i*_).

It should be noted that both models can predict perfect accuracy on the training data. This is because the maximum likelihood separation plane between two Gaussian distributions (as used by the density model) is linear, so the density model is statistically equivalent to a boundary model were all three bounds a learned (i.e., AB, BC, and CD). Hence, because the BC bound is not required at training, the training condition would not allow for distinguishing the computational models. However, the model predictions differ drastically at test. The density model could provide perfect accuracy at test, because it learns the generative distributions (i.e., within–category representation) whereas the boundary model cannot predict above chance performance at test because the BC bound is not learned, and only the bounds that were trained are learned (i.e., between–category representation).

Because the boundary model is a special case of the density model, and the density model has twice as many parameters, the density model is guaranteed to always fit the training data at least as well as the boundary model. To avoid this problem, we estimated the generalization error of the model on the test data for model selection. Specifically, cross–validation [35, 36] was used to identify the type of representations learned by each participant. The following procedure was repeated separately for each participant. First, each model (i.e., Eqs 1, 2 and 4) was fit to the data from the last 200 training trials (Blocks 4–5). These trials were selected because performance is more stable at the end of training (see Fig 3). Next, the generalization error was calculated on the test data (Block 6), without refitting the model parameters (using Eqs 1, 3 and 4). Using the generalization error to select models instead of the training error (as is customary in psychology) avoids the problem of overfitting [34, 35], and actually puts the model with the most free parameters at a disadvantage. If the density model had the smallest generalization error, then it was inferred that the participant learned a within–category representation. If the boundary model had the smallest generalization error, then it was inferred that the participant learned a between–category representation. This fitting procedure was applied individually to each participant in each condition.

#### Model–based results

The root mean square deviation (RMSD) on the generalization error for each subgroup of participants in each training condition is shown in Table 1. As can be seen, there was a large difference in generalization error between the the two models for each group of participants. For the A/B condition, participants best fit by the boundary model (identified as learning between–category representations) had a RMSD for the boundary model that was more than 4 times smaller than the RMSD of the density model. Similarly, participants that were best fit by the density model (identified as learning within–category representations) had a RMSD for the density model that was about 3 times smaller than the RMSD of the boundary model. Similar results were obtained for the Yes/No condition. Hence, the best-fitting model was clearly identified by using the generalization error estimated at test.

**Table 1:** Average root mean square deviation for each model in each condition of Experiment 1.

Best-fitting model	A/B	Yes/No
Between		
Boundary	0.103	0.081
Density	0.468	0.313
Within		
Boundary	0.531	0.533
Density	0.180	0.124

Between = participants identified as learning between–category representations; Within = participants identified as learning within–category representations.

The model fits show that the training methodology successfully biased the kind of knowledge learned by participants. Specifically, 21/31 (67.7%) of the participants in the Yes/No condition were best fit by the density model whereas only 12/30 (40.0%) of the participants were best fit by a density model in the A/B condition. The remaining participants (10/31 and 18/30, respectively) were best fit by the boundary–based model. This difference in proportion of participants best fit by the density–based model (or boundary–based model) in each training condition is statistically significant according to a test of proportions (*Z* = 2.17, *p* < .05), and suggests that a larger proportion of participants in the Yes/No condition learned within–category representations when compared with participants in the A/B condition.

**Fig 4:**
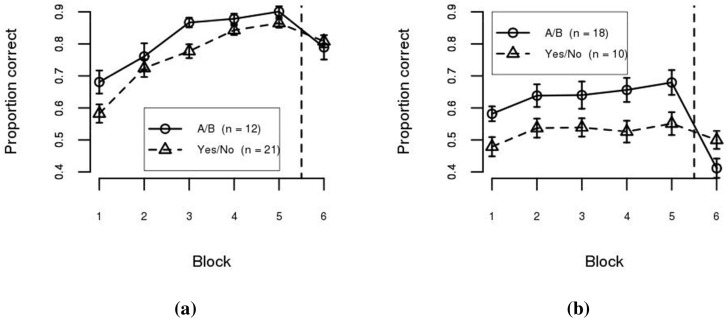
Mean accuracy per block in Experiment 1 separated by best–fitting model. (a) Density–based model. (b) Boundary–based model. Error bars are between–subject standard error of the mean.

The model–based analysis can also be used as a tool to assess the limits of within– and between–category representations for supporting category learning. Fig 4 shows participant accuracy separated by best–fitting model. As can be seen, participants classified as using a within–category representation (i.e., best–fit by the density model—Fig 4a) performed well with both training methodologies. A Condition (A/B, Yes/No) × Training Block (1…5) mixed ANOVA confirmed this observation. The effect of Block was statistically significant (*F*(4, 124) = 55.29, *p* < .001, *η*^2^ = 0.77), but the effect of Condition (*F*(1, 31) = 5.11, *n*.*s*., *η*^2^ = 0.14), and the interaction between the factors (*F*(4, 124) = 1.34, *n*.*s*., *η*^2^ = 0.27), were not statistically significant after application of the Šidák correction for multiple testing. In contrast, participants classified as using a between–category representation (i.e., best–fit by the boundary model—Fig 4b) performed better in the A/B condition than in the Yes/No condition. A Condition (A/B, Yes/No) × Training Block (1…5) mixed ANOVA shows that the effects of Block (*F*(4, 104) = 3.79, *p* < .01, *η*^2^ = 0.37) and Condition were statistically significant (*F*(1, 26) = 6.07, *p* < .05, *η*^2^ = 0.19), but the interaction between the factors was not (*F*(4, 104) = 0.22, *n*.*s*., *η*^2^ = 0.04). These results suggest that a within–category representation can support performance in both the Yes/No and A/B training conditions. However, a between–category representation is more limited, only supporting performance in the A/B condition. Performance at the end of training (Block 5) in the Yes/No condition for participants best fit by the boundary model was only 55.1%, which does not differ from chance performance (*t*(9) = 1.43, *n*.*s*., *η*^2^ = 0.19).

At test, participants best fit by a density–based model performed well (regardless of condition) while participants best fit by a boundary–based model performed poorly (again, regardless of condition). Test performance is not surprising given that the test was specifically designed to require within–category information in order to transfer performance.

### Discussion

Experiment 1 aimed to test the hypothesis that training methodology can bias the kind of representations that are learned with RB structures. Two training methods common in the category–learning literature were used, namely Yes/No (associated with concept learning) and A/B (typical classification instructions). A test phase was designed to assess the ability of participants to contrast new sets of categories using individual categories that were learned during the training phase. This should be possible if within–category representations were learned, but not if between–category representations were learned. The results show that participants in the Yes/No condition could generalize their representations during the test phase (with a small switch cost). This was not the case for participants in the A/B condition, which reverted back to chance performance. Models were fit to identify the type of category representation learned by individual participants. The results show that more participants learned within–category representations with Yes/No training when compared with A/B training. This difference in category representation accounts for participants in the Yes/No condition being able to generalize their knowledge during the test phase, and participants in the A/B condition not being able to generalize their knowledge during the test phase (at the group level).

## Experiment 2

The results of Experiment 1 suggest that training methodology can bias the participants’ category representations, which in turn affects their performance. However, it is unclear whether training methodology would have a similar biasing effect with II structures. Thus, the goal of Experiment 2 was to investigate if training methodology would also affect the nature and generalizability of category representations learned with II structures. Experiment 2 used the same material and experimental procedure as Experiment 1, but the categories were generated such that logical rules were not optimal.

### Method

#### Participants

Fifty-nine participants were recruited from a large state university to participate in this experiment. Participants were randomly assigned to one of two training conditions: Yes/No (*n* = 30) and A/B (*n* = 29). Each participant gave informed written consent and received credit for participation as partial fulfillment of a course requirement. All procedures were approved by the Purdue University Social Science Institutional Review Board. None of the participants in Experiment 2 participated in Experiment 1.

#### Material

**Fig 5:**
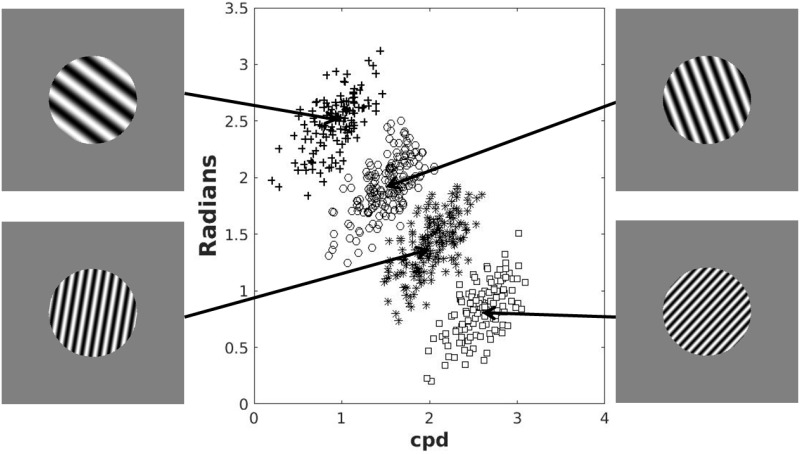
Stimuli used in Experiment 2. The *x*-axis corresponds to the width of the bars and the *y*-axis corresponds to the rotation angle of the bars. Symbols denote different categories. The mean stimulus of each category is shown as an example.

The material was identical to Experiment 1 except for the category structures. The structures used for both training conditions are shown in Fig 5. As in Experiment 1, there were four separate categories generated using the randomization technique [38]. The categories were arbitrarily labeled with letters A–D from top to bottom. Category A stimuli were generated using a bivariate normal distribution with mean *μ*_*A*_ = (0.92, 2.49) and covariance $\Sigma_{A}=(\begin{array}{cc} 0.06 & 0.04\\ 0.04 & 0.06 \end{array})$. Category B stimuli were generated using a bivariate normal distribution with mean *μ*_*B*_ = (1.50, 1.93) and covariance Σ_*B*_ = Σ_*A*_. Category C stimuli were generated using a bivariate normal distribution with mean *μ*_*C*_ = (2.02, 1.41) and covariance Σ_*C*_ = Σ_*A*_. Category D stimuli were generated using a bivariate normal distribution with mean *μ*_*D*_ = (2.59, 0.82) and covariance Σ_*D*_ = Σ_*A*_.

Note that these stimuli have a similar range of *frequency* and *orientation* as those used in Experiment 1 (so they look the same, only the category membership is different). As in Experiment 1, a single set of 600 stimuli was generated from these distributions (shown in Fig 5), and the stimulus set was linearly transformed so that the sample mean and covariance of each category matched the generative distributions. The stimulus set was independently shuffled for each participant. Lastly, as in Experiment 1, these categories can be near–perfectly separated by three boundaries, but in this case the boundaries cannot be described using any obvious logical rule because values on the stimulus dimensions need to be compared and they are not commensurable.

#### Procedure

The procedure was identical to that of Experiment 1.

### Results

**Fig 6:**
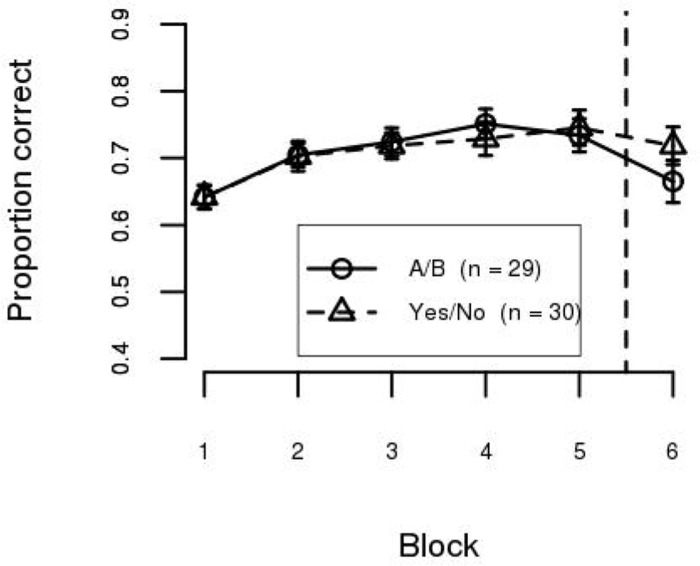
Mean accuracy per block in Experiment 2. Blocks 1–5 are the training phase and Block 6 is the test phase. Error bars are between–subject standard error of the mean.

The mean accuracy for each block in each condition is shown in Fig 6. As can be seen, training accuracy was similar for the A/B and Yes/No training conditions. This was confirmed by a Condition (A/B, Yes/No) × Training Block (1…5) mixed ANOVA. The effect of Block was statistically significant (*F*(4, 228) = 19.04, *p* < .001, *η*^2^ = 0.42) but the effect of Condition (*F*(1, 57) = 0.02, *n*.*s*., *η*^2^ = 0.00) and the interaction between the factors (*F*(4, 228) = 0.44, *n*.*s*., *η*^2^ = 0.04) were not. The mean accuracy in Block 1 was 64.2%, which improved to 74.0% in Block 5, showing that participants in both training conditions learned the task at the same rate. These results are similar to those obtained in Experiment 1.

Unlike in Experiment 1, the accuracy in the test block did not differ between the two training conditions. This was confirmed by a Condition (A/B, Yes/No) × Block (Last training block, Test block) mixed ANOVA. First, unlike in Experiment 1, the Condition × Block interaction did not reach statistical significance (*F*(1, 57) = 1.55, *n*.*s*., *η*^2^ = 0.03). However, similar to Experiment 1, the effect of Block reached statistical significance (*F*(1, 57) = 8.18, *p* < .01, *η*^2^ = 0.13), while the effect of Condition did not (*F*(1, 57) = 0.83, *n*.*s*., *η*^2^ = 0.01). These results suggest a small but statistically significant decrease in accuracy between the last training block and the test block. Accuracy in the test block was reduced to 69.2%, a 4.8% decrease from training accuracy. Hence, both training conditions in Experiment 2 produced results similar to the Yes/No condition from Experiment 1.

#### Model–based results

The same model–based analyses were run as in Experiment 1. As in Experiment 1, the the best-fitting model was clearly identified by using the generalization error estimated at test. The RMSD for each subgroup of participants in each training condition is shown in Table 2. As can be seen, there was a large difference in generalization error between the the two models for each group of participants. For the A/B task, participants best fit by the boundary model (identified as learning between–category representations) had a RMSD for the boundary model that was more than 2.5 times smaller than the RMSD of the density model. Similarly, participants that were best fit by the density model (identified as learning within–category representations) had a RMSD for the density model that was about 3 times smaller than the RMSD of the boundary model. Similar results were obtained for the Yes/No task.

**Table 2:** Average root mean square deviation for each model in each condition of Experiment 2.

Best-fitting model	A/B	Yes/No
Between		
Boundary	0.184	0.120
Density	0.449	0.304
Within		
Boundary	0.462	0.470
Density	0.168	0.095

Between = participants identified as learning between–category representations; Within = participants identified as learning within–category representations.

Unlike the RB task in Experiment 1, however, the training methodology did not affect the kind of knowledge learned by participants. Specifically, 24/30 (80.0%) of the participants in the Yes/No condition were best fit by the density model, while 21/29 (72.4%) of the participants were best fit by a density model in the A/B condition. The remaining participants (6/30 and 8/29, respectively) were best fit by the boundary–based model. This difference in proportion of participants best fit by the density–based model (or boundary–based model) in each training condition is not statistically significant according to a test of proportions (*Z* = 0.68, *n*.*s*.) and suggests that the same proportion of participants learned within–category representations with A/B and Yes/No training.

**Fig 7:**
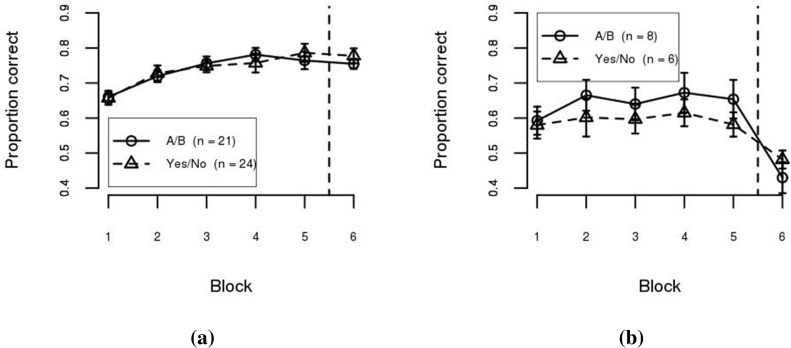
Mean accuracy per block in Experiment 2 separated by best–fitting model. (a) Density–based models. (b) Boundary–based models. Error bars are between–subject standard error of the mean.

Fig 7 shows participant accuracy separated by best–fitting model. Similar to Experiment 1, participants best–fit by the density model (panel a) performed well with both training conditions. A Condition (A/B, Yes/No) × Training Block (1…5) mixed ANOVA confirmed this observation. The effect of Block was statistically significant (*F*(4, 172) = 20.66, *p* < .001, *η*^2^ = 0.53), while the effect of Condition (*F*(1, 43) = 0.00, *n*.*s*., *η*^2^ = 0.00), and the interaction between the factors (*F*(4, 172) = 0.68, *n*.*s*., *η*^2^ = 0.08), were not. However, participants best–fit by the boundary model (panel b) performed poorly in both conditions. A Condition (A/B, Yes/No) × Training Block (1…5) mixed ANOVA shows that the effects of Block (*F*(4, 48) = 1.18, *n*.*s*., *η*^2^ = 0.24) and Condition (*F*(1, 12) = 0.76, *n*.*s*., *η*^2^ = 0.06) were not statistically significant. The interaction between the factors also failed to reach statistical significance (*F*(4, 48) = 0.34, *n*.*s*., *η*^2^ = 0.09). This suggests that learning within–category information is required in order to learn II category structures.

Performance at test was similar to that observed in Experiment 1. Participants best fit by a density–based model performed well (regardless of condition) while participants best fit by a boundary–based model performed poorly (again, regardless of condition). Again, test performance is not surprising given that the test was specifically designed to require within–category information in order to transfer performance.

### Discussion

The goal of Experiment 2 was to test the extent to which training methodology would be an important factor for category structures thought to lead to the development of within–category representations. The results show that training methodology does not affect the type of knowledge learned with II structures. Regardless of training methodology, roughly 75% of the participants learned a within–category representation in Experiment 2. The results of Experiment 2 also suggest that within–category representations are better able to support the learning of II categories (when compared with between–category representations).

## General discussion

This article reports the results of two experiments that investigate the impact of training methodology and category structure on category representation. With the RB category structures of Experiment 1, most participants in classification training (i.e., A/B) learned between–category representations whereas concept training (i.e., Yes/No) biased participants to learn within–category representations. In contrast, with the II structures of Experiment 2, most participants learned within–category representations regardless of training methodology. Importantly, only within–category representations were able to support generalization to the novel categorization problem presented during the test phase. Accuracy–based analyses, as well as analyses derived from the novel model–based approach, jointly support these conclusions.

Classification and concept training are popular training methods in the category learning literature [2, 6, 14–17, 27, 40]. Classification training instructions emphasize learning to distinguish between members of different categories (A/B training; e.g., Is the stimulus a member of category A or category B?). In contrast, concept training instructions emphasize learning about members of each category (Yes/No training; e.g., Is the stimulus a member of category A?). After being trained on two sets of two categories, the participants were tested on a new categorization problem built using the training categories. In Experiment 1, this seemingly subtle variation in training methodology had a profound impact on performance with RB category structures. Participants in the Yes/No condition were successful in generalizing their learned category representations at test, only demonstrating a very minor cost in accuracy. Participants in the A/B condition, however, had the opposite result, generally showing little–to–no evidence of generalization.

The accuracy–based analyses of Experiment 1 are consistent with the claim that classification training results in between–category representations and that concept training results in within–category representations. What was necessary, however, was a technique that would allow for the identification of the learned representation and, importantly, an assessment of the extent to which the learned representation can support generalization. To accomplish this, computational models with different representational assumptions were fit to individual participant data—as is often done in category learning (e.g., [4]). The utility of this approach was enhanced by the application of a technique from the machine learning literature (i.e., cross–validation [34, 35], see also [36]). More specifically, models assuming pattern classification or density estimation [20], as representatives of between– or within–category representations (respectively), were fit to the training data of individual participants. The model parameters were fixed and used to predict the generalization error during the test phase for each participant. Participants were assumed to have used the representation included in the model with the smallest generalization error. This novel application of the computational approach provided two important insights in Experiment 1. First, training methodology does not guarantee a particular type of representation (e.g., 40% of the participants in the A/B condition used within–category representations). Second, the ability to generalize between–category representations to support a reconfiguration of knowledge appears to be quite limited (e.g., between–category representations were at a disadvantage during training and test—Fig 4b).

Experiment 2 investigated the impact of training methodology on II category structures—which are thought to promote within–category representations [23, 26, 27]. Training methodology had no impact on learning. This results was unexpected given that Yes/No training has been shown to impair II learning [14]. In stark contrast to Experiment 1, training methodology also had no impact on generalizability. The model–based analysis suggests that this was due to the vast majority of participants learning within–category representations, which are better suited for generalization in the current test phase, regardless of the training task.

### Utility of between– and within–category representations

The distinction between within– and between–category representations is not new and has been implicitly present in category learning models for over 30 years. For example, prototype [8] and exemplar [9] approaches have used within–category representations, while criterion setting models (e.g., [10, 11]) have been using between–category representations. However, the focus on this critical representational difference is more recent [5, 19]. Because prototype and exemplar models use within–category representations, they would predict good transfer performance at test in the present experiment, while criterion setting models would have predicted poor transfer (because they use between–category representations). However, none of these models naturally predicts the effect of training methodology in Experiment 1, or the participant individual differences in knowledge representation observed in both experiments. A hybrid categorization model would more naturally account for the results obtained in the experiments included in this article (e.g., [3, 13]).

In addition, our results suggest that, at least for the current paradigm, within–category representations may be the default outcome of category learning (which may explain the continued success of prototype and exemplar models). Between–category representations may be limited to contexts in which there is a match between the representational bias provided by the category structure and training methodology. In other words, between–category representations may be the exception rather than the rule.

How can this issue be reconciled with the prominence of rule–based approaches in theories of category learning (e.g., [3, 13, 41, 42])? Indeed, many empirical studies have argued that humans (and non–human primates) have a bias to use logical rules [24, 43–46]. Two considerations are in order. First, both between– and within–category representations can serve as the building blocks for logical rules. For example, knowing that the stimuli from one category are large and the stimuli from a contrasting category are small can be used to instantiate a logical rule derived from within–category representations. Likewise, knowing the density used to represent each category (as in the proposed density–based model) allows for the calculation of the best separation plane (boundary). However, the boundary is not explicitly represented in the density–based model, just like the logical rule is not directly represented in the within–category representation above (i.e., large/small). Second, our experimental and analytical approach focused on the outcome of learning rather than the beginning of learning. Thus, the results do not preclude an initial bias toward between–category representations that may eventually shift to within–category representations in most contexts. However, the results do suggest that within–category representations may be the most common outcome of category learning.

The results also suggest that within–category representations may have greater utility than between–category representations. Participants using within–category representations were better equipped to generalize their knowledge to support test phase performance. It is important to note that successful generalization required participants to reconfigure the knowledge acquired during training. More specifically, participants had to categorize familiar stimuli/categories in a novel way. This may not be a trivial issue as previous work suggests that knowledge generalization may be superior for between–category representations when the test phase utilizes novel stimuli, but the categorization rule is familiar [23]. For example, knowing that red fruits taste better than green fruits (a between–category representation) may be more useful than knowing that red fruits are good and that green fruits are good (both within–category representations) when faced with a choice between a new red or a new green fruit. However, the between–category representation about fruit taste does not tell us anything about the actual taste of red and green fruits (e.g., they could both be bad, with green fruits being worse than red fruits). Hoffman and Rehder refer to this phenomenon as the “cost of supervised classification” [47]. Using a similar paradigm to ours, they showed that classification (A/B) training can lead to selective attention, thus learning to ignore stimulus dimensions that may end up being relevant in future contexts, and limiting generalizability. In the present article, we show that even if the new context requires knowledge of the learned stimulus dimension, there is a cost to classification learning if the new context requires decisions in a new part of the stimulus dimensional–space. To summarize, it may be that within–category representations favor generalization depending on the reconfiguration of current knowledge in a novel way whereas between–category representations favor generalization depending on the application of current knowledge to novel stimuli.

### Training methodology and knowledge representation

One important implication of the experiments in this article is the impact of training methodology on knowledge representation. Participants learn what is required by the task, and slightly changing the task requirements may affect the knowledge representation that is learned. The learned representation affects how the knowledge can be used. For example, Chin–Parker and Ross showed that training participants to infer a missing stimulus feature (given the category label and some of the stimulus features) promotes the learning of within–category representations [33] (see also [48]). Likewise, Levering and Kurtz have shown that observational learning leads to knowledge consistent with generative models [7] (also within–category representation). We have shown in this article that asking participants to separate stimuli into a number of categories (i.e., classification training) leads most participants to learn between–category representations if simple logical rule are optimal, but not if such rules fail. In the latter case, participants learn within–category representations (similar to inference and observational training). In contrast, training participants to learn about category members (i.e., concept training) promotes learning within–category representations for both RB and II category structures. Both classification learning and concept learning tend to be used interchangeably in the categorization literature, but these results suggest that they should not.

Differences between what is learned with each training methodology may have been missed in past research because categorization tasks are rarely followed by a test phase that is not simply composed of new stimuli to be put in the training categories. However, these differences in categorical representations are critical because category learning outside of the laboratory is rarely the ultimate goal [2, 5, 31, 49]. Outside of the laboratory, animals typically learn to categorize in order to facilitate interactions with their environment. For example, by categorizing a pet as a cat, one knows a lot about the animal without necessarily having to interact with it (e.g., it likes to sleep, it is small and fury, etc). Given that categorization is often an intermediate step towards a greater goal, the type of category representation is critical in determining how useful it will be in various situations. Intuitively, learning within–category representations appears to be more useful in most cases. For example, learning within–category representations of cats and dogs is more useful than exclusively learning a between–category representation between cats and dogs. Knowing what a cat is will facilitate distinguishing a cat from a ferret, whereas only learning what is different between cats and dogs may not help with the ferret (for a similar argument, see [47]).

### Limitations

The experiments included in this article explore the conditions leading to the formation of within– or between–category representations using a transfer test that can only be performed if participants learn to distinguish all pairs of trained categories. If only the contrasts included at training are learned, test performance should be poor. We argued that successful test performance was possible if participants learned within–category representations, but not between–category representation. The underlying assumption is that participants learning within–category representations would succeed at test because they would have learned about all the categories at training (because they were all part of some of the contrasts), but that participants who learned between–category representations would fail at test because they would only learn bounds required for the training contrasts (i.e., AB and CD, but not BC). However, it is possible that participants learn the BC bound during training, even though they are never asked to contrast these categories. We argue that this possibility is unlikely because (1) participants typically learn only the information that is required to perform the task [5, 50] and (2) the participants never receive feedback about the BC bound, which would make the criterion difficult to learn [51]. Still this possibility is not completely ruled out by the current experiments.

Another limitation of the current article is that the boundary–based model seems to pick–up mostly participants that performed poorly at training. This is interesting because model selection relied on generalization error. Both models could predict perfect performance at training. However, only the density model could predict good performance at test, whereas both the density and boundary model could predict poor performance at test. The boundary–based model had an advantage over the density–based model since (1) both models could predict perfect training accuracy and (2) the boundary–based model is nested in the density–based model (so it should avoid overfitting). As a result, it is expected that participants performing well at test would be best fit by the density model (the test was designed to rely on within–category representations), and these participants likely also did well at training (it would be difficult for a participant to successfully transfer knowledge that was poorly acquired). While both models would be able to account for participants who did not perform well at test, the boundary model probably fit best because it avoided overfitting the training data. Future work should explore designing a new test condition where between–category knowledge would predict better transfer to confirm that the models correctly identify the content of the participants’ knowledge representation.

Finally, Experiments 1 and 2 used RB and II category structures (respectively) because these are well–studied categories [52]. However, another difference between the experiments is that Experiment 1 required selectively attending to only one stimulus dimension, whereas Experiment 2 required attending to both stimulus dimensions. Hence, it is possible that the difference between the results obtained in the two experiments depends on attention rather than the RB/II distinction. Specifically, this would mean that unidimensional categorization could produce between– or within–category representations depending on the training condition, whereas categorization using multiple features would produce within–category representations, regardless of training methodology. This article does not attempt to distinguish between these two possibilities. The conclusions reached in this article do not depend on the RB vs. II distinction, and whether the interaction between training condition and category structures depends on the RB/II distinction or attentional effects is not the focus of the current article.

### Conclusion and future work

The ecological advantage of within–category representations may explain why the default type of knowledge learned by participants, at least in the conditions tested in this article, is within–category representation. The only exception was A/B training with a RB structure, and this training condition only reduced the probability of learning within–category representations to about 0.4. Hence, this training method may be more artificial and less ecologically valid. Future research should focus on testing the ecological validity of category learning experiments using this methodology. Also, work in this article suggests that identifying the type of representations learned by participants is critical in understanding how the knowledge can be used in different tasks. Given that categorization outside the laboratory is often an intermediate step towards a greater goal, more category learning experiments should include a variety of transfer tasks to test the robustness and generality of the acquired knowledge. This may allow for improving training regiments in different training programs.

## Supporting information

S1 File “S1_File.zip”. This file contains the raw data from the experiments reported in this manuscript.
(ZIP)

## References

[1] Markman AB. Stimulus categorization In: Pashler H & Medin DL, editor. Stevens’ Handbook of Experimental Psychology. vol. 2: Memory and cognitive processes. 3rd ed New York: John Wiley & Sons; 2002 p. 165–208.

[2] Helie S, Ashby FG. Learning and transfer of category knowledge in an indirect categorization task. Psychological Research. 2012;76:292–303. 10.1007/s00426-011-0348-1 21660482

[3] Ashby FG, Alfonso-Reese LA, Turken AU, Waldron EM. A neuropsychological theory of multiple systems in category learning. Psychological Review. 1998;105(3):442 10.1037/0033-295X.105.3.442 9697427

[4] Maddox WT, Ashby FG. Comparing decision bound and exemplar models of categorization. Perception & Psychophysics. 1993;53(1):49–70. 10.3758/BF03211715 8433906

[5] Markman AB, Ross B. Category use and category learning. Psychological Bulletin. 2003;129:529–613. 10.1037/0033-2909.129.4.592 12848222

[6] Smith JD, Minda JP. Distinguishing prototype-based and exemplar-based processes in dot-pattern category learning. Journal of Experimental Psychology: Learning, Memory, & Cognition. 2002;28:800–811. 12109770

[7] Levering KR, Kurtz KJ. Observation versus classification in supervised category learning. Memory & Cognition. 2015;43(2):266–282. 10.3758/s13421-014-0458-2 25190494

[8] Reed SK. Pattern recognition and categorization. Cognitive Psychology. 1972;3:382–407. 10.1016/0010-0285(72)90014-X

[9] Nosofsky RM. Attention, similarity, and the identification-categorization relationship. Journal of Experimental Psychology: General. 1986;115:39–57. 10.1037/0096-3445.115.1.39 2937873

[10] Erev I. Signal detection by human observers: A cutoff reinforcement learning model of categorization and decisions under uncertainty. Psychological Review. 1998;105:280–298. 9669925 10.1037/0033-295x.105.2.280

[11] Treisman M, Williams TC. A theory of criterion setting with an application to sequential dependencies. Psychological Review. 1984;91:68–111. 10.1037/0033-295X.91.1.68

[12] Hammer R, Diesendruck G, Weinshall D, Hochstein S. The development of category learning strategies: What makes the difference? Cognition. 2009;112(1):105–119. 19426967 10.1016/j.cognition.2009.03.012

[13] Nosofsky RM, Palmeri TJ, McKinley SC. Rule-plus-exception model of classification learning. Psychological review. 1994;101(1):53 10.1037/0033-295X.101.1.53 8121960

[14] Maddox WT, Bohil CJ, Ing AD. Evidence for a procedural-learning-based system in perceptual category learning. Psychonomic Bulletin & Review. 2004;11(5):945–952. 10.3758/BF03196726 15732708

[15] Posner MI, Keele SW. On the genesis of abstract ideas. Journal of Experimental Psychology. 1968;77:353–363. 10.1037/h0025953 5665566

[16] Reber PJ, Stark CE, Squire LR. Cortical areas supporting category learning identified using functional MRI. Proceedings of the Academy of Sciences. 1998;95:474–750. 10.1073/pnas.95.2.747 PMC18492 9435264

[17] Zeithamova D, Maddox WT, Schnyer DM. Dissociable prototype learning systems: evidence from brain imaging and behavior. Journal of Neuroscience. 2008;28:13194–13201. 10.1523/JNEUROSCI.2915-08.2008 19052210 PMC2605650

[18] Casale MB, Ashby FG. A role for the perceptual representation memory system in category learning. Perception & Psychophysics. 2008;70:983–999. 10.3758/PP.70.6.983 18717385 PMC2562695

[19] Carvalho PF, Goldstone RL. The benefits of interleaved and blocked study: Different tasks benefit from different schedules of study. Psychonomic Bulletin & Review. 2015;22:281–288. 10.3758/s13423-014-0676-4 24984923

[20] Bishop C. Pattern Recognition and Machine Learning. Singapore: Springer; 2006.

[21] Muggleton S. Inductive Logic Programming. Singapore: Academic Press; 1992.

[22] Ashby FG, Ell SW. The neurobiology of human category learning. Trends in Cognitive Science. 2001;5:204–210. 10.1016/S1364-6613(00)01624-7 11323265

[23] Casale MB, Roeder JL, Ashby FG. Analogical transfer in perceptual categorization. Memory & Cognition. 2012;40:434–449. 10.3758/s13421-011-0154-4 22183985

[24] Ell SW, Ashby FG. The impact of category separation on unsupervised categorization. Attention, Perception, & Psychophysics. 2012;74:466–475. 10.3758/s13414-011-0238-z 22069083

[25] Ell SW, Ing AD, Maddox WT. Critrial noise effects on rule-based category learning: The impact of delayed feedback. Attention, Perception, & Psychophysics. 2009;71(6):1263–1275. 10.3758/APP.71.6.1263 PMC2730042 19633342

[26] Ashby FG, Waldron EM. On the nature of implicit categorization. Psychonomic Bulletin & Review. 1999;6(3):363–378. 10.3758/BF03210826 12198775

[27] Thomas RD. Learning correlations in categorization tasks using large, ill-defined categories. Journal of Experimental Psychology: Learning, Memory, & Cognition. 1998;24:119–143. 10.1037//0278-7393.24.1.119 9438955

[28] Ashby FG, Crossley MJ. A computational model of how cholinergic interneurons protect striatal-dependent learning. Journal of Cognitive Neuroscience. 2011;23(6):1549–1566. 10.1162/jocn.2010.21523 20521851

[29] Helie S, Ell SW, Filoteo JV, Maddox WT. Neural mechanisms of criterion learning in rule-based categorization: A new model. Brain and Cognition. 2015;95:19–34. 25682349 10.1016/j.bandc.2015.01.009 PMC4385499

[30] Anderson JR. The Adaptive Character of Thought. Hillsdale, NJ: Erlbaum; 1990.

[31] Minda JP, Ross BH. Learning categories by making predictions: An investigation of indirect category learning. Memory & Cognition. 2004;32:1355–1368. 10.3758/BF03206326 15900929

[32] Maddox WT, Filoteo JV, Lauritzen JS, Connally E, Hejl KD. Discontinuous categories affect information-integration but not rule-based category learning. Journal of Experimental Psychology: Learning, Memory, & Cognition. 2005;31:654–669. 10.1037/0278-7393.31.4.654 16060771

[33] Chin-Parker S, Ross BH. The effect of category learning on sensitivity to within-category correlations. Memory & Cognition. 2002;30:353–362. 10.3758/BF03194936 12061756

[34] Hastie T, Tibshirani R, Friedman J. The elements of statistical learning: Data mining, inference, and prediction. New York: Springer; 2001.

[35] Helie S. An introduction to model selection. Tutorials in Quantitative Methods for Psychology. 2006;2:1–10. 10.20982/tqmp.02.1.p001

[36] Busemeyer JR, Wang YM. Model comparisons and model selections based on generalization criterion methodology. Journal of Mathemtical Psychology. 2000;44(1):171–189. 10.1006/jmps.1999.1282 10733863

[37] Brainard DH. The psychophysics toolbox. Spatial Vision. 1997;10:433–436. 10.1163/156856897X00357 9176952

[38] Ashby FG, Gott RE. Decision rules in the perception and categorization of multidimensional stimuli. Journal of Experimental Psychology: Learning, Memory, and Cognition. 1988;14:33–53. 2963894 10.1037//0278-7393.14.1.33

[39] Ashby FG. Multivariate probability distributions In: Ashby F, editor. Multidimensional Models of Perception and Cognition. Hillsdale, NJ: Erlbaum; 1992.

[40] Ashby FG, Maddox WT. Stimulus categorization In: Birnbaum M, editor. Measurement, judgment, and decision making: Handbook of perception and cognition. San Diego: Academic Press; 1998.

[41] Erickson MA, Kruschke JK. Rules and exemplars in category learning. Journal of Experimental Psychology: General. 1998;127(2):107 10.1037/0096-3445.127.2.107 9622910

[42] Shepard RN, Hovland CI, Jenkins HM. Learning and memorization of classifications. Psychological Monographs: General and Applied. 1961;75:1–42. 10.1037/h0093825

[43] Ashby FG, Queller S, Berretty PM. On the dominance of unidimensional rules in unsupervised categorization. Perception & Psychophysics. 1999;61:1178–1199. 10.3758/BF03207622 10497436

[44] Ell SW, Ashby FG. The effects of category overlap on information-integration and rule-based category learning. Perception & Psychophysics. 2006;68(6):1013–1026. 10.3758/BF03193362 17153195

[45] Medin DL, Wattenmaker WD, Hampson SE. Family resemblance, conceptual cohesiveness, and category construction. Cognitive Psychology. 1987;19:242–279. 10.1016/0010-0285(87)90012-0 3581758

[46] Smith JD, Beran MJ, Crossley MJ, Boomer JT, Ashby FG. Implicit and explicit category learning by macaques (Macaca mulatta) and humans (Homo sapiens). Journal of Experimental Psychology: Animal Behavior Processes. 2010;36:54–65. 10.1037/a0015892 20141317 PMC2841782

[47] Hoffman AB, Rehder B. The costs of supervised classification: The effect of learning task on conceptual flexibility. Journal of Experimental Psychology: General. 2010;139(2):319–340. 10.1037/a0019042 20438254

[48] Yamauchi T, Markman AB. Category learning by inference and classification. Journal of Memory and Language. 1998;39:124–148. 10.1006/jmla.1998.2566

[49] Brooks LR, Squire-Graydon R, Wood TJ. Diversion of attention in everyday concept learning: Identification in the service of use. Memory & Cognition. 2007;35:1–14. 10.3758/BF03195937 17533875

[50] Pothos EM, Chater N. A simplicity principle in unsupervised human categorization. Cognitive Science. 2002;26:303–343. 10.1207/s15516709cog2603_6

[51] Hélie S, Ell SW, Filoteo JV, Maddox WT. Criterion learning in rule-based categorization: Simulation of neural mechanism and new data. Brain and cognition. 2015;95(1):19–34. 10.1016/j.bandc.2015.01.009 25682349 PMC4385499

[52] Maddox WT, Ashby FG. Dissociating explicit and procedural–learning based systems of perceptual category learning. Behavioural Processes. 2004;66:309–332. 10.1016/j.beproc.2004.03.011 15157979

